# Supplementary figures and images for: Weighted gene co-expression network analysis reveals key genes and lncRNAs in Bos indicus chronically infected with Johne’s disease
Source: Front Immunol. 2026 Jun 3;17:1754171. doi: 10.3389/fimmu.2026.1754171 (PMC13271941; doi:10.3389/fimmu.2026.1754171)

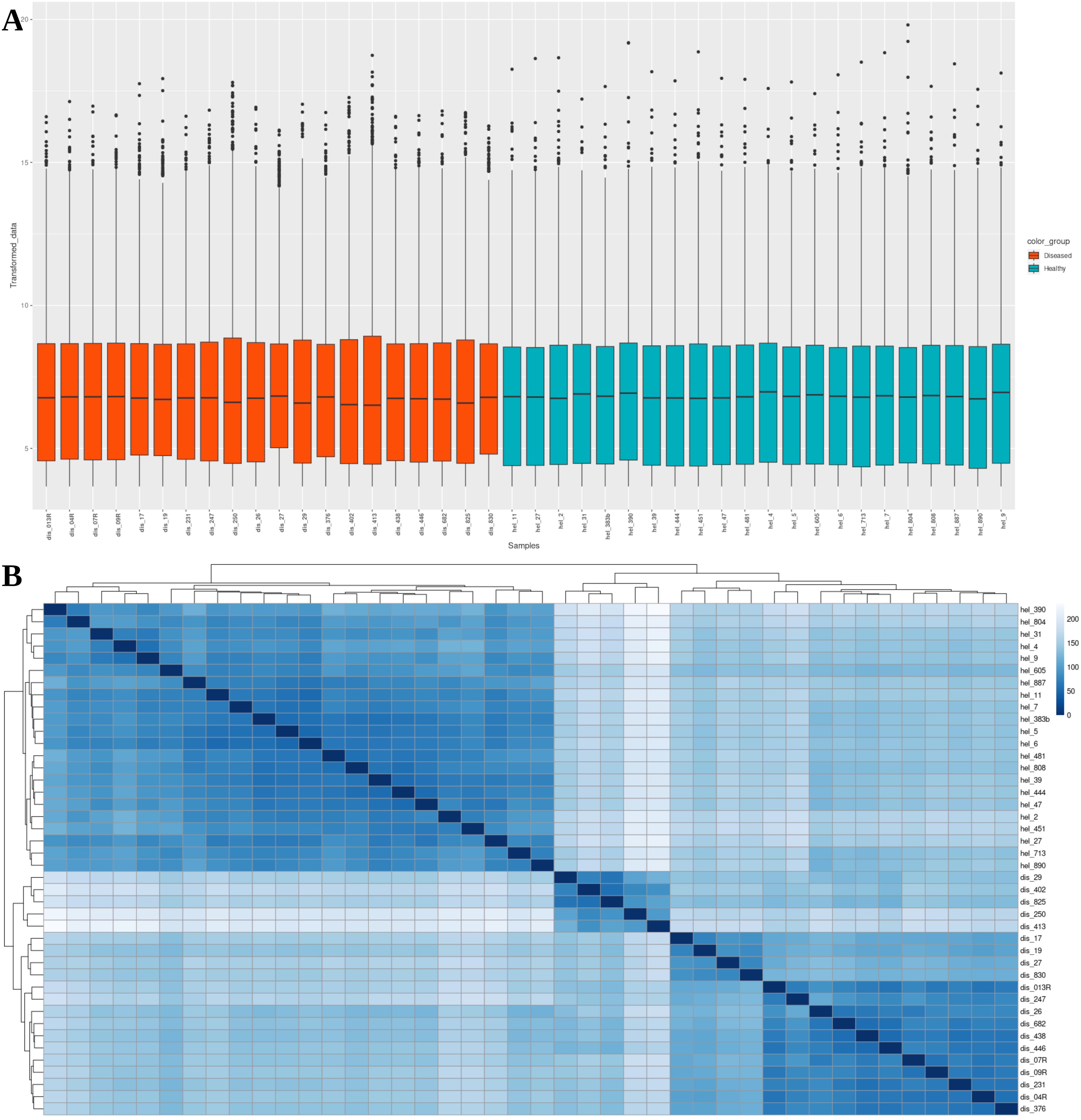

Supplement: Supplementary Figure 1 — Data normalization and sample clustering. (A) Boxplot of transformed and normalized counts for each sample, illustrating consistent distribution across samples. (B) Hierarchical clustering dendrogram of samples based on gene expression patterns, showing distinct clusters that separate diseased and healthy samples. [file Image1.tif]

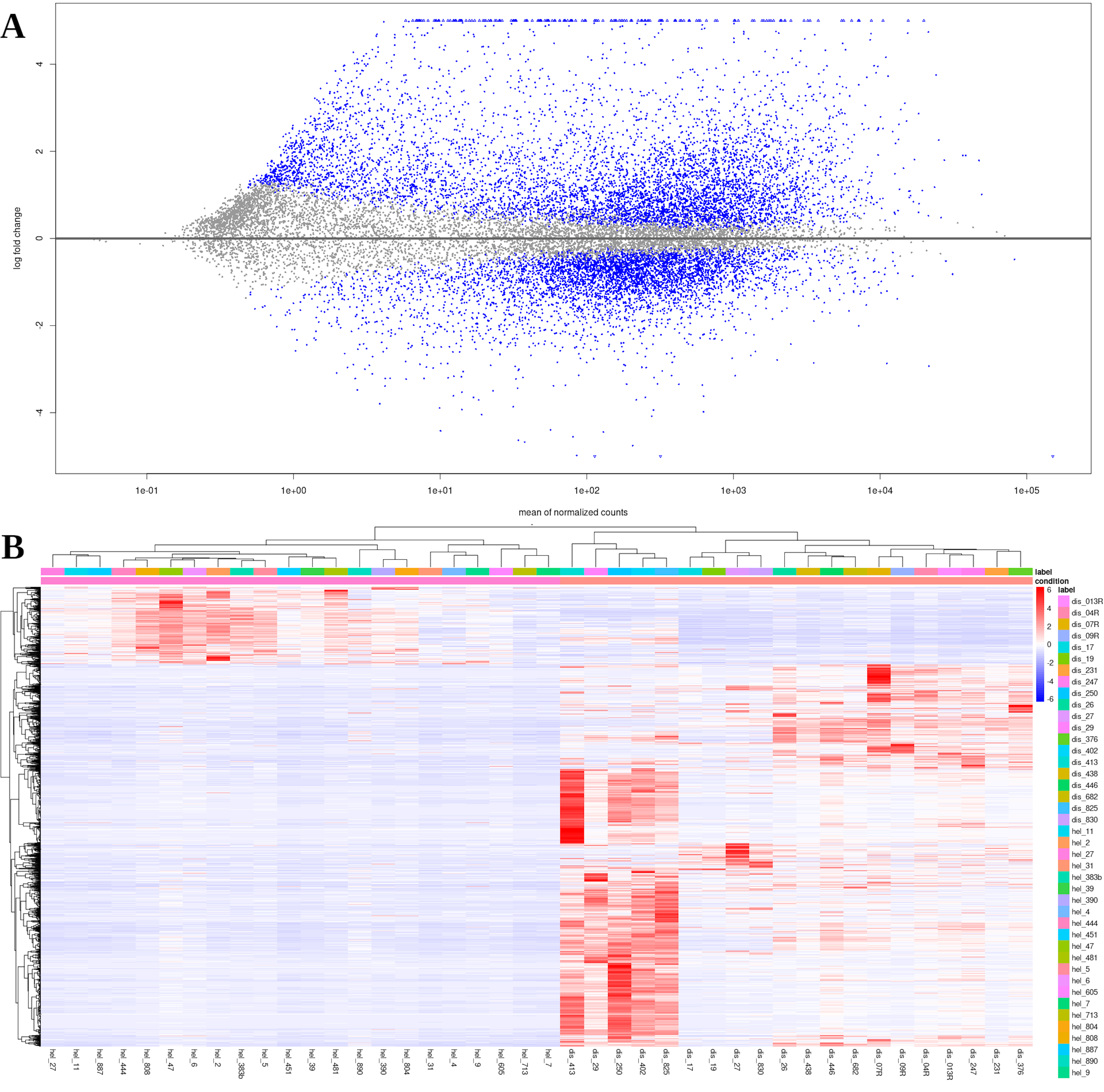

Supplement: Supplementary Figure 2 — Differential expression of mRNA. (A) MA plot showing the relationship between the average expression (log-transformed mean counts) and the log2 fold change of differentially expressed mRNAs between disease and healthy conditions. (B) Heatmap displaying the expression patterns of differentially expressed genes across disease and healthy samples. Each row represents a gene, and each column represents a sample. [file Image2.tif]

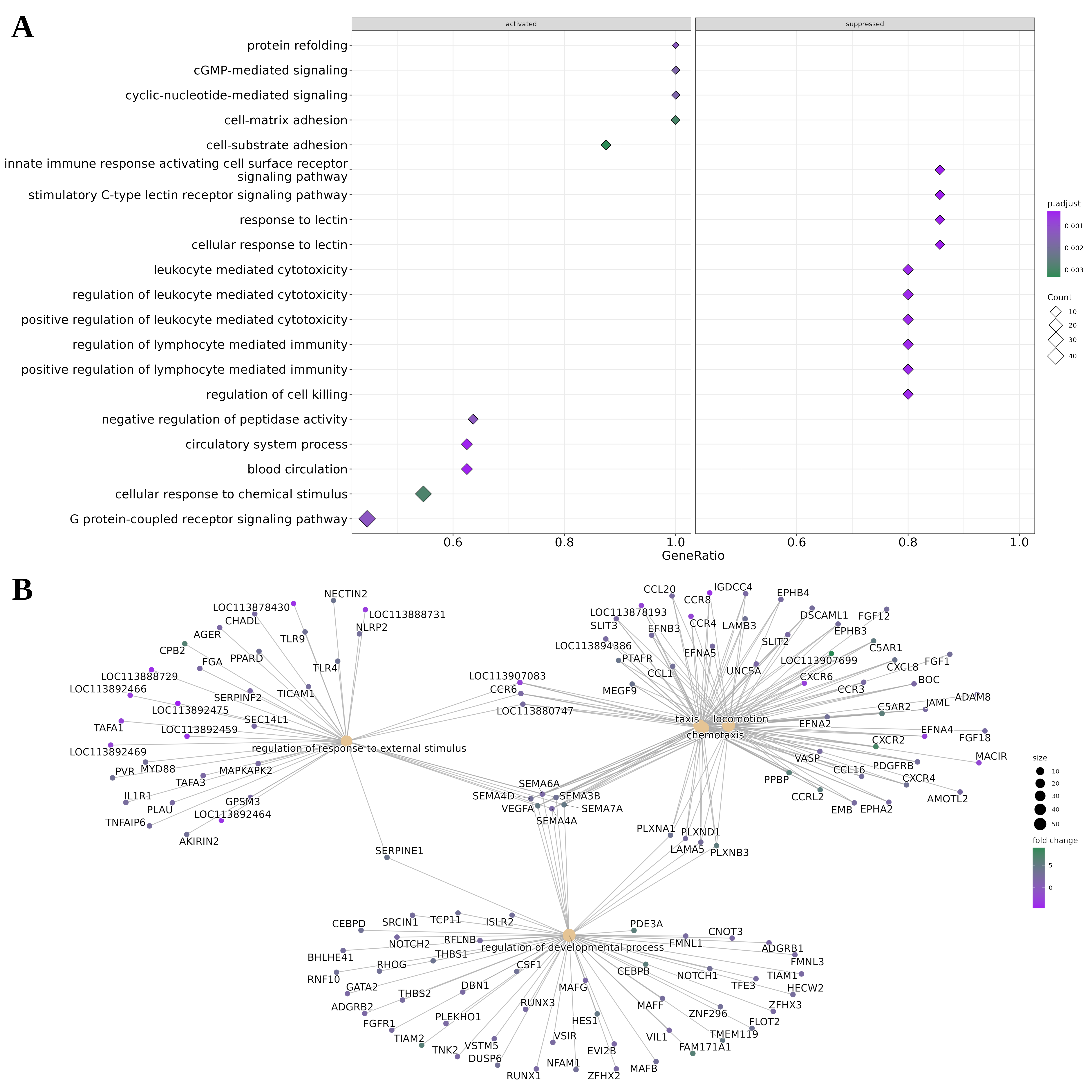

Supplement: Supplementary Figure 3 — Enrichment of differentially expressed genes. (A) GSE plot displaying the top 20 pathways activated or suppressed in response to MAP infection. (B) Cnet plot showing the top five enriched GO terms and the genes associated with BP. [file Image3.tif]

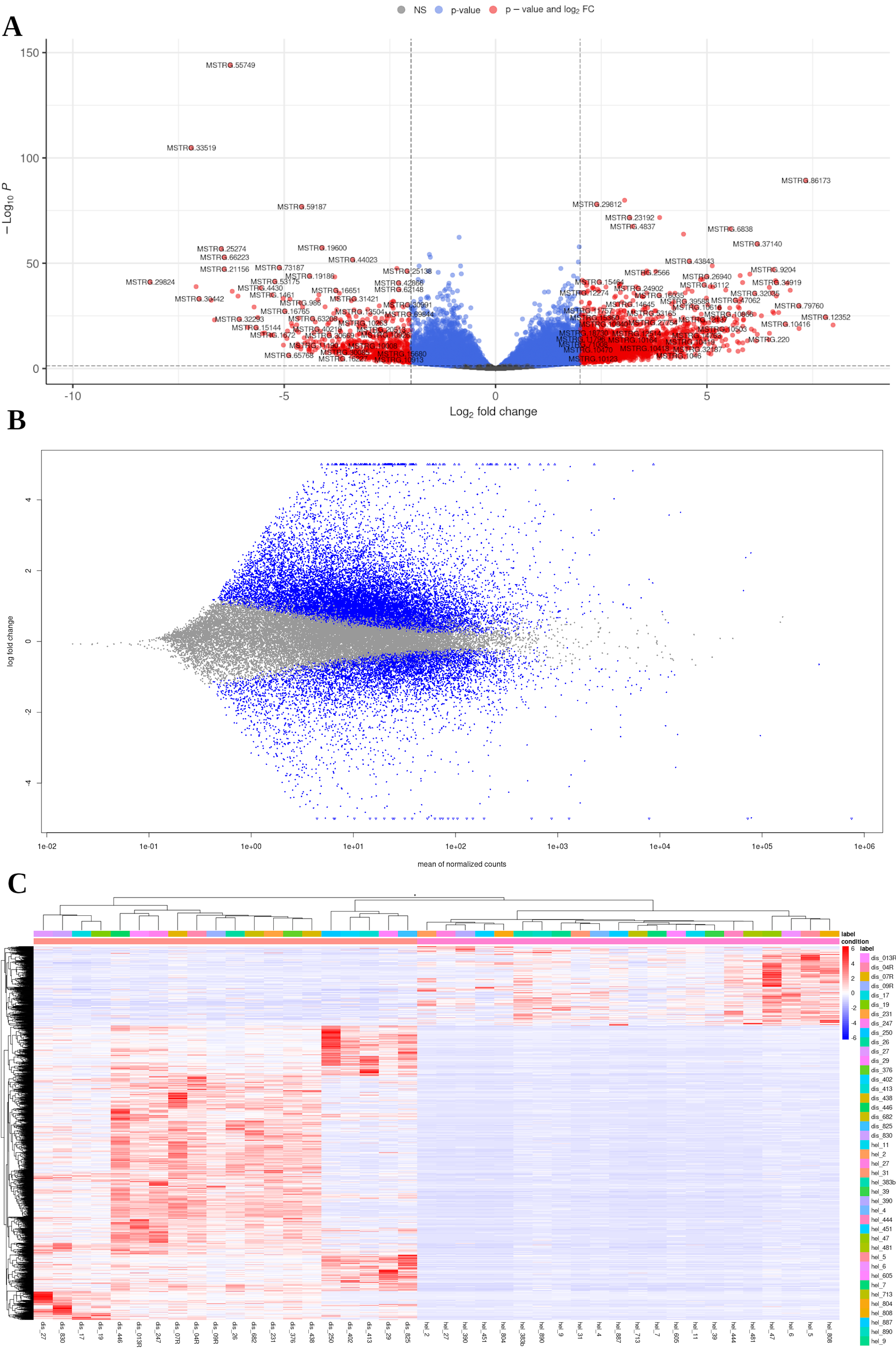

Supplement: Supplementary Figure 4 — Differential expression of lncRNAs. (A) Volcano plot showing differentially expressed lncRNAs, with red points indicating upregulated and downregulated genes based on the log2 fold change (log2(FC)) between disease and healthy samples. (B) MA plot displaying the relationship between average expression (log-transformed mean counts) and the log2 fold change of differentially expressed lncRNAs between disease and healthy conditions. (C) Heatmap of differentially expressed lncRNAs in healthy versus diseased samples. [file Image4.tif]

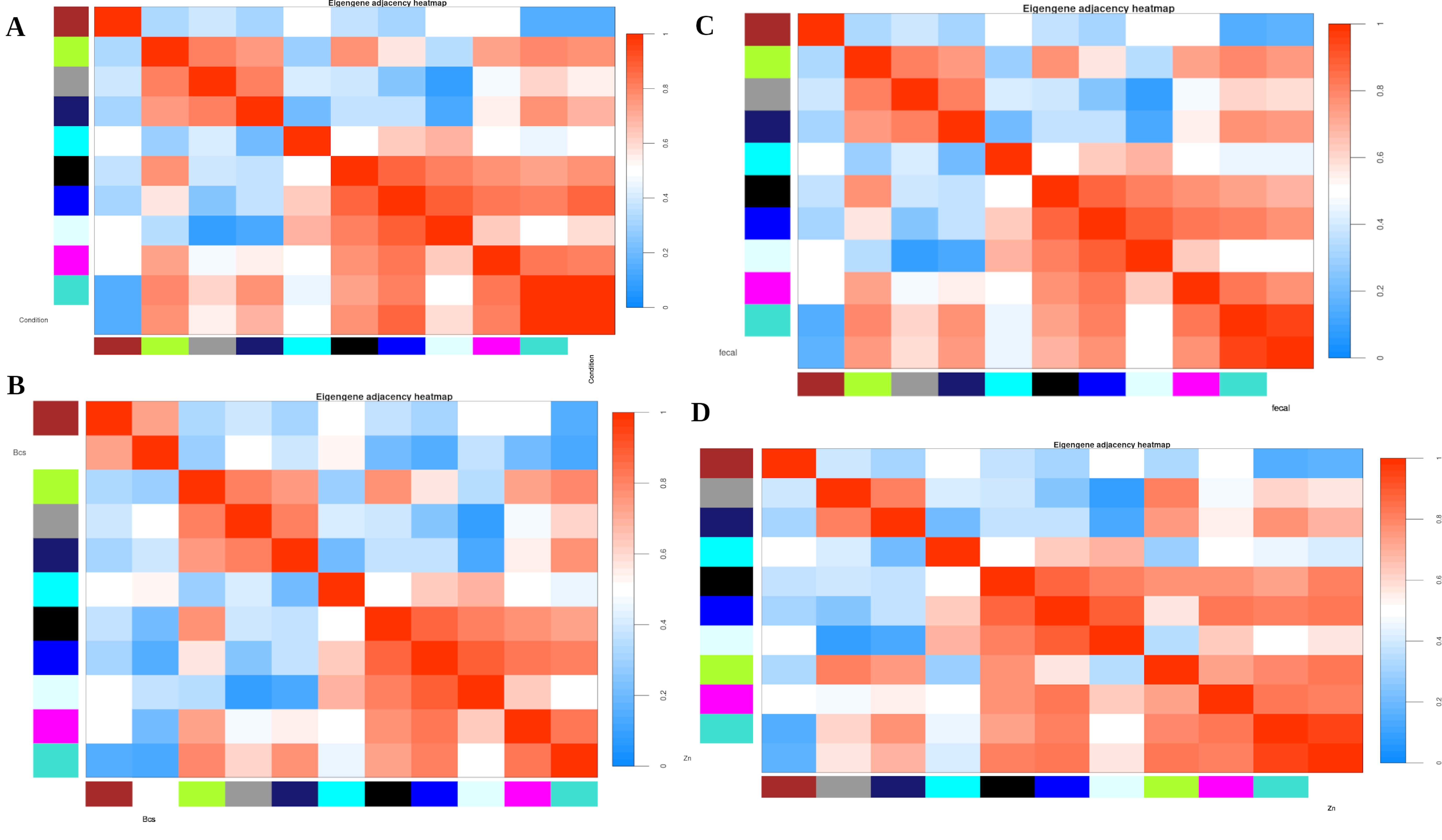

Supplement: Supplementary Figure 5 — Eigengene heatmap showing correlated modules with traits. Heatmap of module eigengenes to identify clusters of correlated eigengenes (modules) in relation to each trait. (A) Condition (B) BCS (C) Fecal (D) ZN staining. [file Image5.tif]

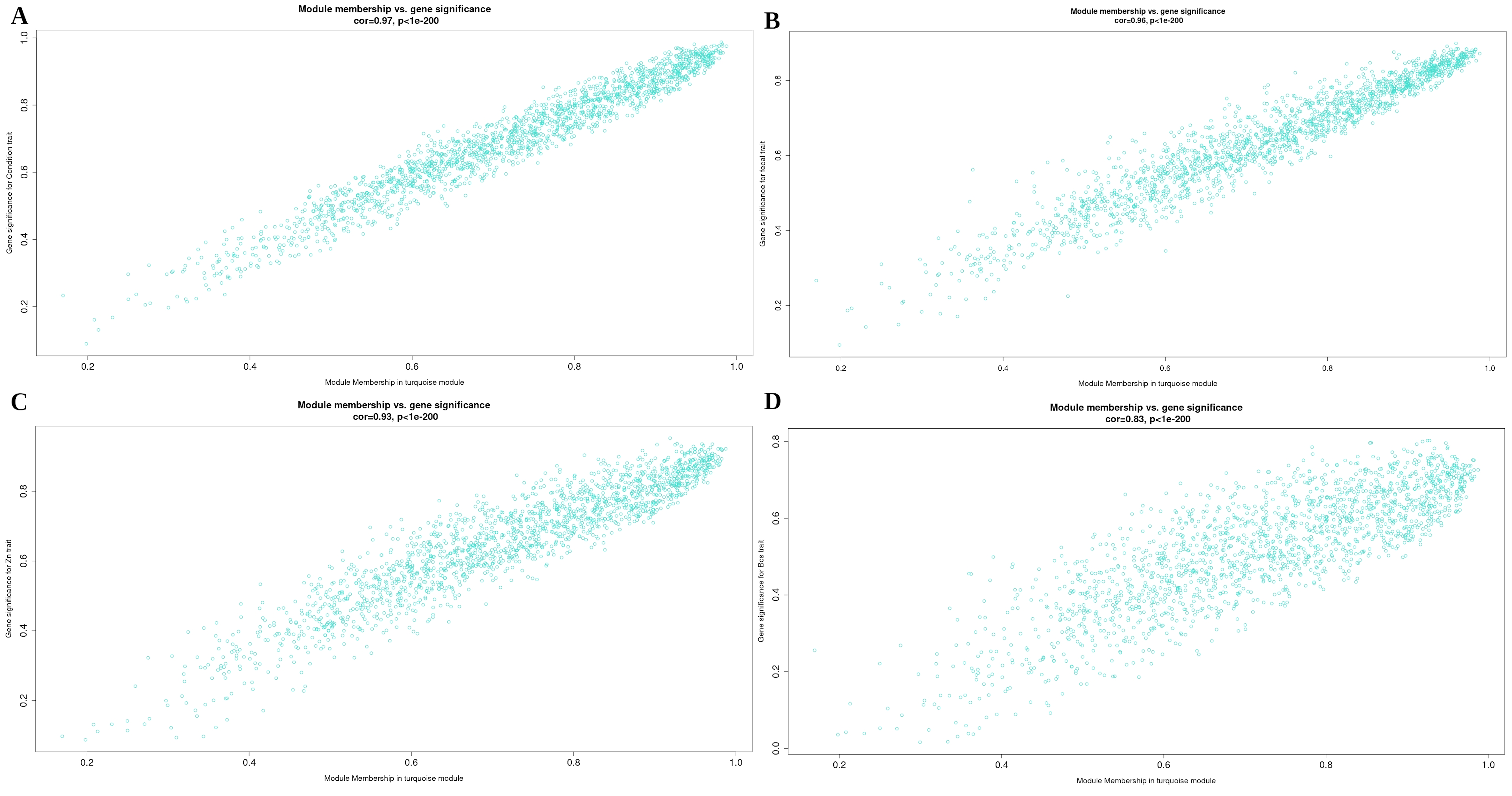

Supplement: Supplementary Figure 6 — Heatmap of MM and GS in the turquoise module. (A) MM vs. GS with condition trait (B) MM vs. GS with fecal trait (C) MM vs. GS with ZN staining trait (D) MM vs. GS with BCS trait. [file Image6.tif]

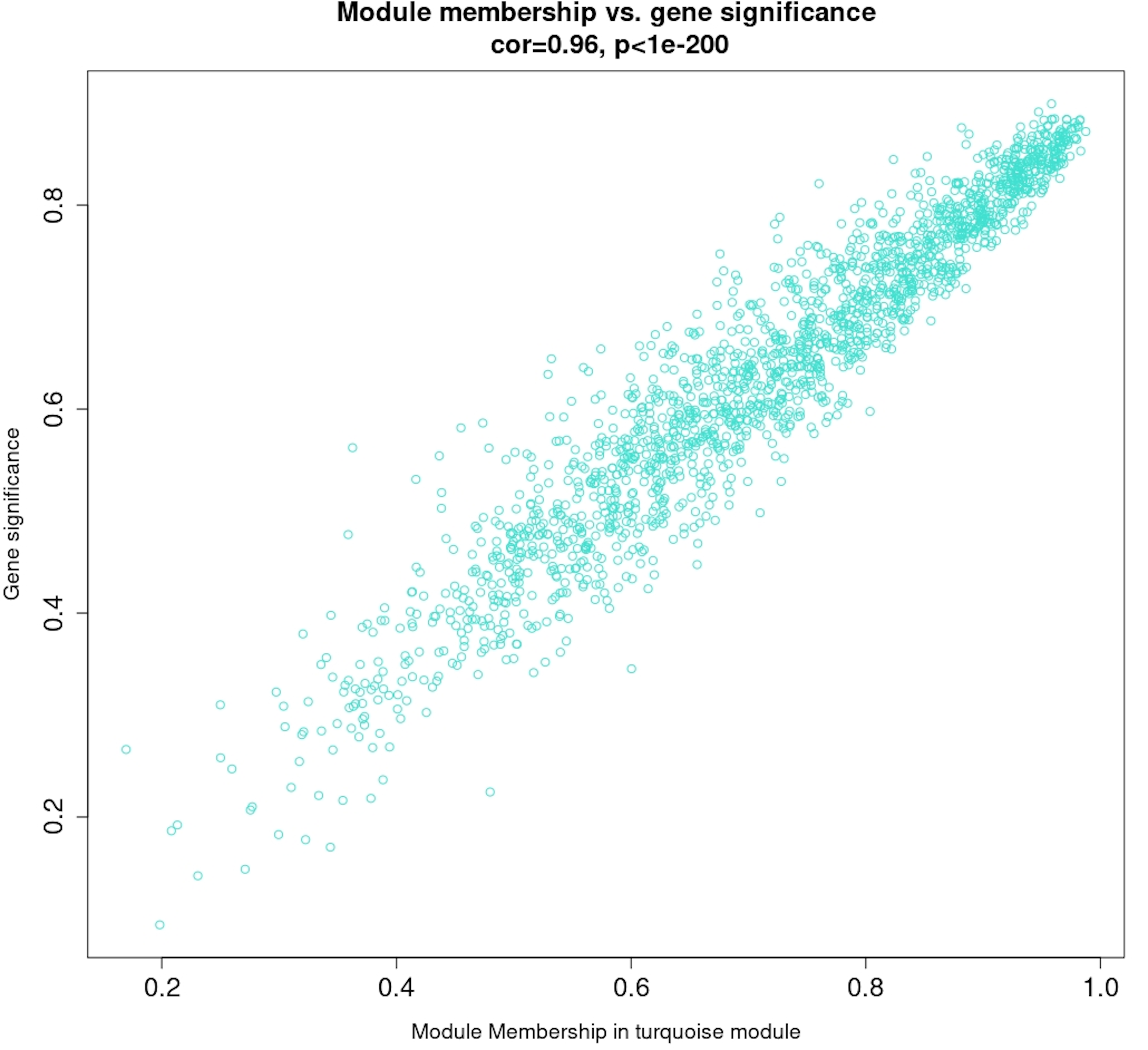

Supplement: Supplementary Figure 7 — Relationship between MM and GS for MAP infection in turquoise module. Scatter plot showing the correlation between MM and GS in the turquoise module for MAP infection, comparing diseased and healthy cattle. [file Image7.tif]

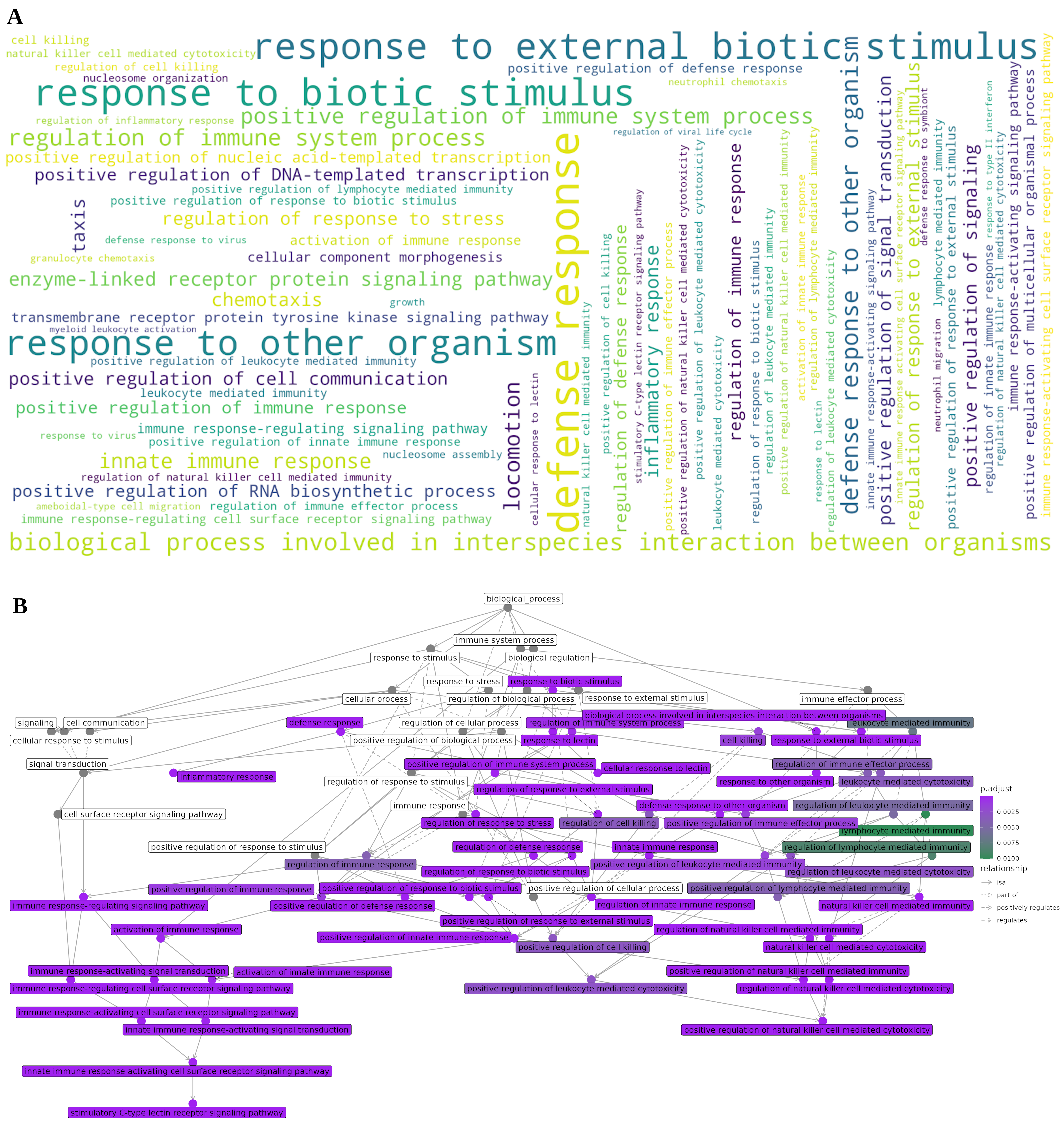

Supplement: Supplementary Figure 8 — GO enrichment visualization of protein coding genes in turquoise module. (A) Word cloud of all enriched GO BP terms, with font size representing the frequency and significance of biological processes associated with protein coding genes. Larger terms indicate higher enrichment significance. (B) Enriched GO network graph showing the relationships among the top GO terms in BP. Nodes represent enriched GO terms, with edges indicating functional relationships based on shared genes. Node size reflects significance, and color intensity represents gene count. This graph highlights interconnected pathways. [file Image8.tif]

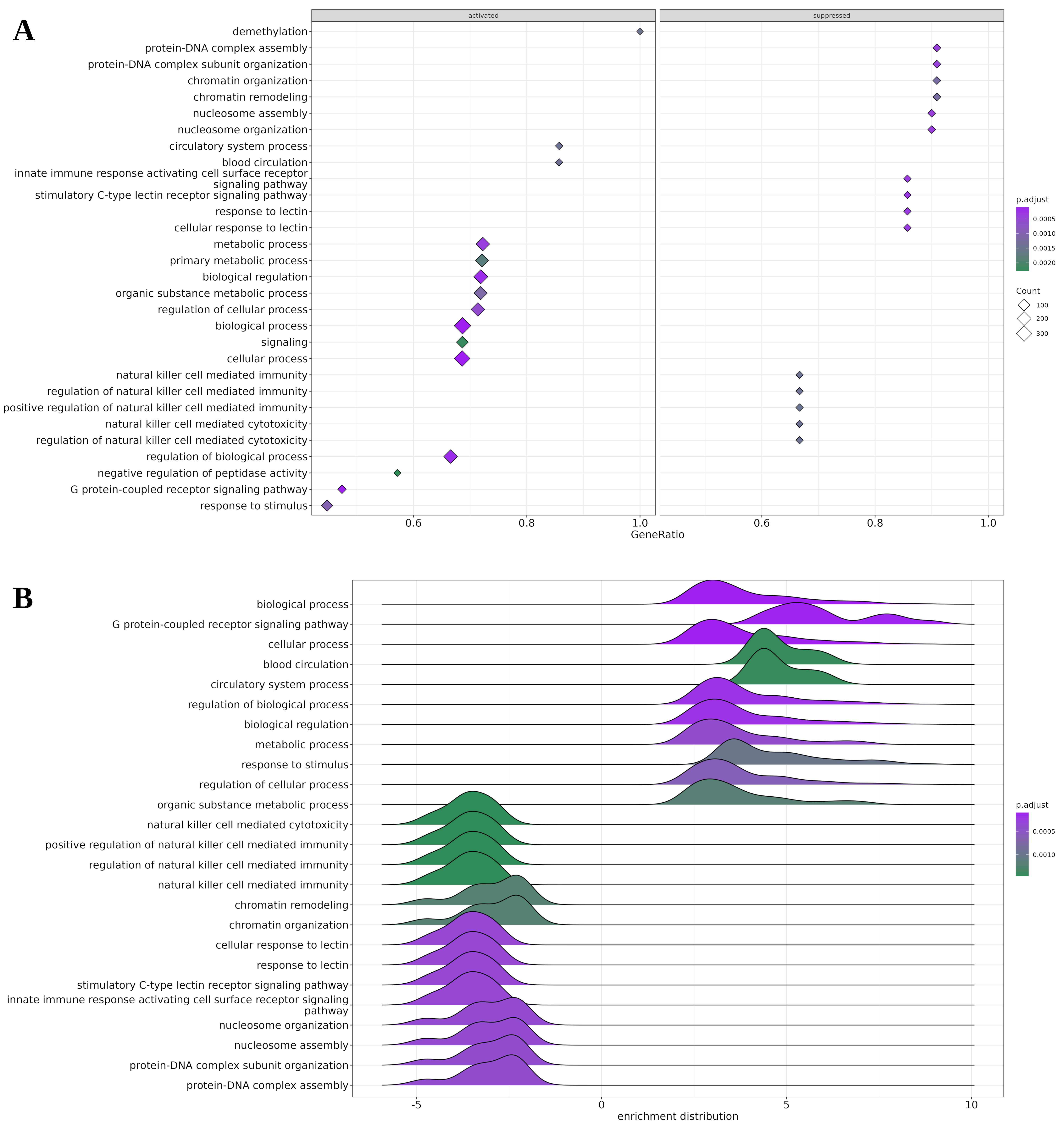

Supplement: Supplementary Figure 9 — Gene set enrichment analysis. (A) GSE plot illustrating the top 30 enriched pathways, highlighting those that are activated or suppressed in the dataset. (B) Ridge plot showing the distribution of the top 25 upregulated and downregulated pathways, providing insight into the variability and significance of pathway changes. [file Image9.tif]

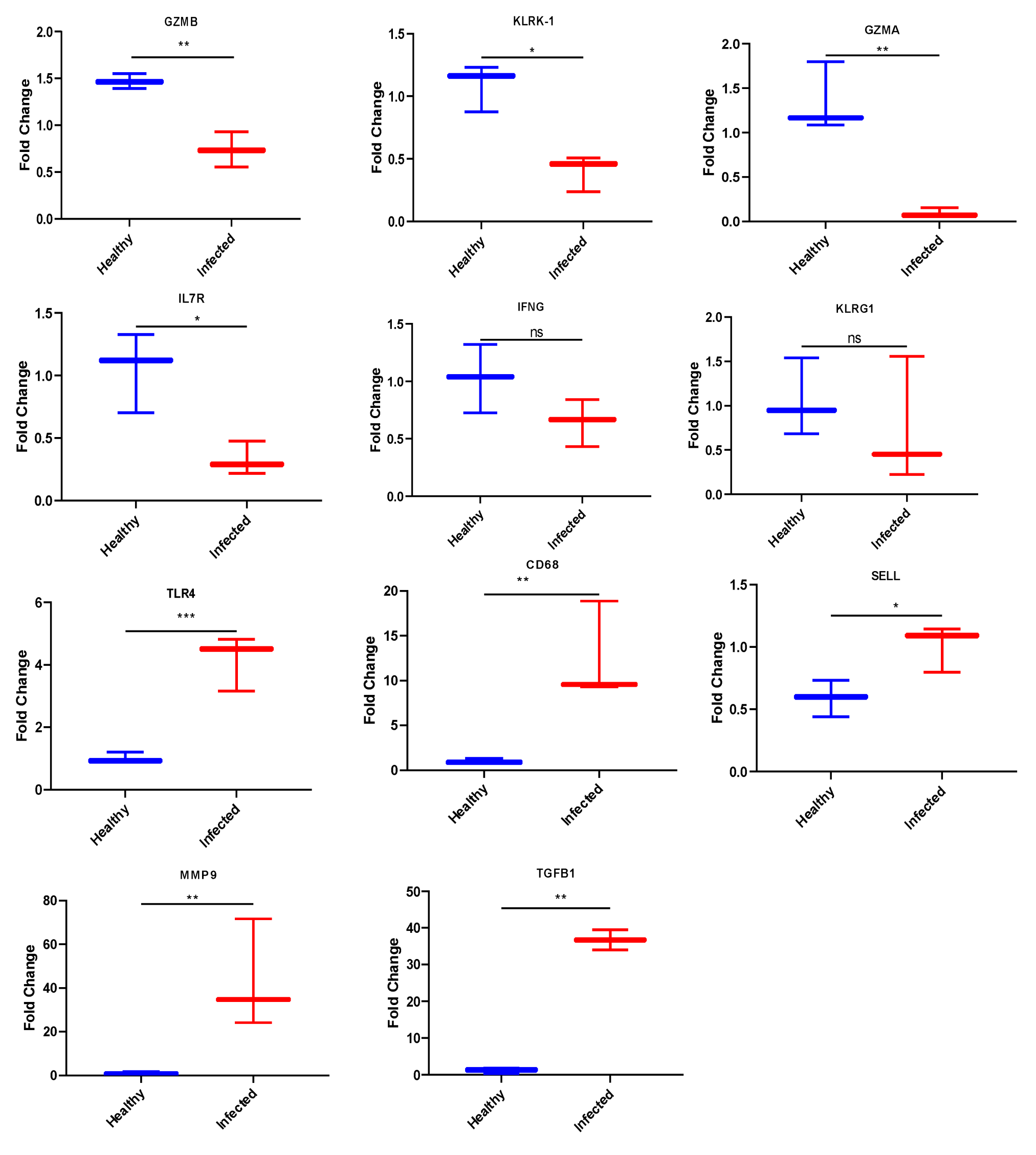

Supplement: Supplementary Figure 10 — Experimental validation of in silico-identified hub genes by RT-qPCR. Box plots display the expression levels of hub genes in healthy versus diseased conditions. Statistical significance was assessed using an unpaired Student’s t-test (*P < 0.05, **P < 0.01, ***P < 0.001, ns = non-significant). [file Image10.tif]
